# G1 checkpoint establishment *in vivo* during embryonic liver development

**DOI:** 10.1186/1471-213X-14-23

**Published:** 2014-05-19

**Authors:** Xiao Qi Wang, Kwok Kin Chan, Xiaoyan Ming, Vincent CH Lui, Randy YC Poon, Chung Mau Lo, Chris Norbury, Ronnie TP Poon

**Affiliations:** 1 Department of Surgery, The University of Hong Kong, 21 Sassoon Road, Pokfulam, Hong Kong, China; 2 State Key Laboratory for Liver Research, The University of Hong Kong, Pokfulam, Hong Kong, China; 3 Division of Life Science, University of Science & Technology, Clear Water Bay, Hong Kong, China; 4 Sir William Dunn School of Pathology, University of Oxford, Oxford, UK

**Keywords:** Embryonic liver, Cell cycle checkpoint, Ionizing radiation

## Abstract

**Background:**

The DNA damage-mediated cell cycle checkpoint is an essential mechanism in the DNA damage response (DDR). During embryonic development, the characteristics of cell cycle and DNA damage checkpoint evolve from an extremely short G1 cell phase and lacking G1 checkpoint to lengthening G1 phase and the establishment of the G1 checkpoint. However, the regulatory mechanisms governing these transitions are not well understood. In this study, pregnant mice were exposed to ionizing radiation (IR) to induce DNA damage at different embryonic stages; the kinetics and mechanisms of the establishment of DNA damage-mediated G1 checkpoint in embryonic liver were investigated.

**Results:**

We found that the G2 cell cycle arrest was the first response to DNA damage in early developmental stages. Starting at E13.5/E15.5, IR mediated inhibition of the G1 to S phase transition became evident. Concomitantly, IR induced the robust expression of p21 and suppressed Cdk2/cyclin E activity, which might involve in the initiation of G1 checkpoint. The established G1 cell cycle checkpoint, in combination with an enhanced DNA repair capacity at E15.5, displayed biologically protective effects of repairing DNA double-strand breaks (DSBs) and reducing apoptosis in the short term as well as reducing chromosome deletion and breakage in the long term.

**Conclusion:**

Our study is the first to demonstrate the establishment of the DNA damage-mediated G1 cell cycle checkpoint in liver cells during embryogenesis and its *in vivo* biological effects during embryonic liver development.

## Background

Endogenous and exogenous DNA damage are life-long threats to the health of an organism and can limit the survival and regenerative potential of both embryonic stem cells (ESCs) and adult stem cells (ASCs). Any genetic alterations in the progenitor cells can compromise the genomic stability and functionality of entire cell lineages [1-3]. Moreover, damage to cellular DNA can be the most important initiating factor in the development of cancer. The DNA damage response (DDR) network has developed to sense and respond to DNA damage and is critical for the maintenance of genetic integrity. The DDR is a complex network that involves the control of cell cycle arrest, the activation of DNA repair machinery, the induction of apoptosis, and the regulation of telomere length. Within this network, the activation of DNA damage checkpoints plays a central role in DDR signaling to ensure the correct scan of the entire genome before DNA replication (S phase) and cell division (M phase) [2,3].

Although the DNA damage-mediated checkpoint is critical in DDR signaling, many of the regulatory components that govern this signaling pathway, specifically in cells at the embryonic stage and during developmental processes, are not known. The cell cycle and the DNA damage checkpoint change over time, from an extremely short G1 cell cycle to maintain pluripotency, to lengthening the G1 phase during differentiation [4-7]; from lacking a G1 checkpoint [8-10] to the establishment of a G1 cell cycle arrest. For example, both murine and human ES cells, as well as human embryonic carcinoma (EC) cells, are defective in the G1 checkpoint after DNA damage [8-10]. Differentiated EC cells show an increased G1 cell population but lack a G1 checkpoint, even though DDR protein activation appears to be normal [9]. Thus, the developmental stages and circumstances under which the G1 cell cycle checkpoint is required remain unclear. Moreover, most studies regarding DDR checkpoints and DNA repair in human and murine stem cells have been performed in vitro with ESC lines and compared to mouse embryonic fibroblasts (MEFs) or genetic knockout MEFs, an approach which reflects only a short period of embryonic development.

For stem or progenitor cells, it is necessary to evolve effective and non-mutagenic DNA repair capacities to avoid passing mutations on to subsequent generations and initiating cancers [11,1]. In both humans and mice, ES cells have been shown to be more capable of repairing DNA damage than their differentiated derivatives [2]. Recent studies suggest that the kinetics of DNA repair are different in the hematopoietic stem cells (HSCs) and progenitor cells of human versus mouse; whereas murine HSCs display faster repair kinetics, human HSCs are less capable of DNA repair and are more pro-apoptotic [1,11,12]. Therefore, different cell lineages, as well as different species of stem and progenitor cells, have different DDR and repair capacities. DNA double-strand breaks (DSBs), which arise during DNA replication or following exposure to ionizing radiation (IR), are considered the most harmful lesions. The principal mechanisms of DSB repair in mammalian cells include nonhomologous end-joining (NHEJ) and homologous recombination repair (HR). HR ensures accurate DSB repair, while NHEJ repair is rapid and efficient but error-prone [13,14].

In the past, MEF cells, derived from various genetic knockout mice, have been used for cell cycle related studies *in vitro* whereas cell cycle studies *in vivo* at embryonic stages have been performed by in situ assays. There have been no detailed investigations of DDR kinetics, including checkpoints and DNA damage repair, at different embryonic developmental stages during organ development by using live cells. In this study, we investigated when (at which embryonic stage) and how the DNA damage-mediated G1 checkpoint is established during *in vivo* embryonic liver development and associated DNA damage repair pathways.

## Methods

### Mouse strains and embryos

ICR mice (CD-1, Harlan UK Ltd, UK) were provided and maintained by the Laboratory Animal Unit of the University of Hong Kong and used for all experiments. Embryos at different stages, including E11.5, E13.5, E15.5, and E17.5, were obtained from pregnant ICR mice. Post-natal mice at P0, P7, P14, P21, and P56 were also used. H&E stained mouse liver tissue structures from embryonic stage to adult were shown in Additional file 1: Figure S1. This study was approved by The Committee on the Use of Live Animals of the University of Hong Kong (CULATR 1623–08).

### Ionizing radiation (IR)

Pregnant mice were subjected to 4–6 Gy of IR (Gammacell 3000, MDS Nordion, Germany) at defined embryonic stages. At 0, 6, 16, and 24 hours after IR, pregnant mice were sacrificed, and embryonic livers were dissected for cell cycle analysis and other experiments. P0 to P56 mice were also subjected to 2 Gy of IR, and the liver cells were isolated at multiple time points.

### Isolation of fetal or adult liver cells

Fetal livers were dissected out from mouse embryos (E11.5 liver had to be dissected out under a dissection microscope), minced, and digested with collagenase-V (100 units/ml, Sigma-Aldrich, St. Louis, MO, USA) for 10 minutes at 37°C. The tissue was then filtered through a 40 μm nylon mesh to remove debris. The cells were collected by centrifugation (500 g for 5 minutes) at 4°C. Isolated single liver cells were fixed with cold 80% ethanol and kept at -20°C for cell cycle analysis. The same procedure was used to isolate adult liver cells. For cell cycle analysis, a pool of 3–5 of E11.5 embryonic livers and 2–3 of E13.5 or E15.5 fetal livers was collected.

### Tissue specimens and nuclear protein fractions

The liver tissue was frozen in liquid nitrogen immediately after harvest for the generation of protein lysates. For nuclear protein extraction, 20 g of fresh fetal liver was homogenized thoroughly on ice and centrifuged. The pellet was re-suspended in buffer B (5 mM HEPES, 1.5 mM MgCl_2_, 0.2 mM EDTA, 0.5 mM DTT, 26% (v/v) glycerol, pH 7.9) and 300 mM NaCl for 20 minutes at 4°C. After centrifugation (24000 g for 20 minutes at 4°C), the supernatant containing the nuclear protein was kept at -70°C prior to performing the DNA repair assays. For immunohistochemistry, liver tissue was fixed in 4% paraformaldehyde overnight and then embedded in paraffin blocks. For E11.5 to E15.5, the whole embryos were fixed; for E17.5 to P56, the dissected livers were fixed.

### Flow cytometry

For analysis of the cell cycle, the nuclei were stained with propidium iodide (PI; Sigma) and analyzed with a Cytomics FC 500 (Beckman Coulter, Indianapolis, IN, USA). Fetal liver cells were also stained with an anti-albumin antibody (R&D Systems, Minneapolis, MN, USA) at different embryonic stages to establish a threshold by which albumin-positive populations were gated for the analysis of DNA content. Starting at E11.5, over 80% of the isolated embryonic liver cells expressed albumin (Additional file 1: Figure S2). The percentage of each cell cycle population was calculated with ModFit v3.1 software (Verity Software House, Topsham, ME, USA).

### Antibodies, Western Blots (WB), and Immunoprecipitation (IP)

Antibodies against cyclins A, E, and B1 and those against Cdk1, p21, RAD51, and Ligase IV were obtained from Santa Cruz Biotechnology (Santa Cruz, CA, USA). Antibodies against cyclin D1, phospho-Cdk2 (Thr160), and γ-H2AX were purchased from Cell Signaling Technology (Beverly, MA, USA). An anti-Cdk2 antibody was kindly provided by Prof. K Yamashita (Kanazawa University, Japan). For WB, 10–40 μg of total tissue protein lysate was loaded and separated on 10% or 12% acrylamide gels and transferred to PVDF membranes, which were then incubated with primary and secondary antibodies. Protein expression was revealed with enhanced chemi lumescent (ECL) reagents (Amersham, GE healthcare, UK). For IP, total cell lysates (100–200 μg) were incubated with 1–2 μl of primary antibody followed by incubation with 30 μl of protein G sepharose (Amersham Biosciences, Sweden). After 3 to 4 washes, the immune complexes were dissolved in SDS sample buffer for WB. For most IP assays, rabbit-derived antibodies were used to avoid cross-reaction.

### Immunohistochemistry

For antigen unmasking, deparaffinized sections were boiled for 10 minutes. After blocking with 3% H_2_O_2_ for 10 minutes and 1% BSA for 1 hour, the sections were incubated with an anti-γH2AX antibody overnight at 4°C. Polymer horseradishperoxidase-conjugated secondary antibodies and DAB + Chromogen (Dako North America, Carpinteria, CA, USA) were used to visualize the signal.

### *In vitro* NHEJ assay

A pUC19 plasmid was cut with the restriction enzyme PvuII overnight at 37°C and followed by alkaline phosphatase (New England BioLabs, Ipswich, MA, USA) treatment for 1 hour to prevent self-ligation of the fragment. The linearized plasmid was identified by agarose gel electrophoresis and purified by a Gel purification kit (Qiagen). 50 ng of the linearized DNA was used as a substrate and incubated with fetal liver nuclear protein (10 μg) in T4 ligase buffer and 1 mM dNTPs for 2 hours at 14°C. After this reaction, the DNA was de-proteinized by purification using a PCR purification kit (Qiagen). The amount of end-joined DNA products was measured by quantitative real-time PCR with primer set A for a loading control, resulting in the amplification of a 151 bp DNA product at a 143 bp distance from a PvuII site. Primer set B flanked the joining junction for the amplification of the joined DNA fragments. The relative NHEJ activity was calculated as the ratio of the end-joined products normalized to the loading control products. Primer sets A and B are shown in Additional file 1: Table S1.

### *In situ* cell death detection

Apoptotic liver cells on embryonic liver sections were determined by using in situ cell death detection fluorescein kit (Roche Applied Science, IN, USA) following manufacturer’s instruction.

### G-banding and spectral karyotyping (SKY) analysis

Pregnant mice were exposed to 0.5 Gy IR at embryonic stages 11.5 and 15.5. The embryos surviving this low dose of IR developed normally. The offspring were allowed to grow to adulthood (7 weeks old), and the liver cells were then isolated from the mice and cultured. Chromosome G-banding and SKY analysis was performed by the Van Andel Institute (Grand Rapids, MI, USA) to examine chromosome breakage and rearrangement.

## Results

### Cell cycle shifting at different developmental stages

**Figure 1:**
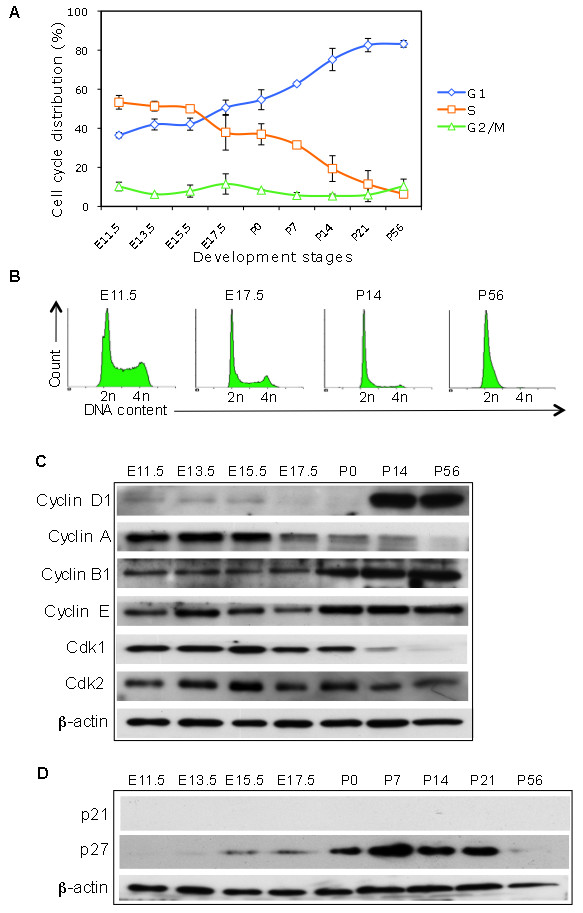
**S and G1 cell cycle distributions in murine liver cells at different stages of development. (A)** Single liver cells were isolated from mice at the embryonic stages E11.5, E13.5, E15.5, and E17.5 and at postnatal days 0, 7, 14, 21 and 56, and were then fixed. The DNA content of PI-stained nuclei was determined by flow cytometry. The cells in the G1, S, and G2/M phases were analyzed (Modfit software) and graphed to determine the distribution pattern during murine development. The mean ± SD was from 2 independent experiments. The S-phase cells were dominant at early stages of development. Starting at E17.5, the G1 population started to increase while the S population decreased. **(B)** Representative histograms of the cell cycle distribution of murine liver tissue at indicated stages. In adult mice (P56), most liver cells were in the G1 cell phase. **(C)** Liver tissue lysates from mice at the indicated stages of development were prepared for WB analysis of Cdk1, Cdk2 and Cdc25A, and the cyclins D1, E, A, and B1 with β-actin as the loading control. **(D)** WB analysis of the Cdk inhibitors p21 and p27.

We dissected and isolated single liver cells from embryonic, postnatal, and adult mice and determined the cell phase by measuring the PI-stained DNA content. From E11.5 to E15.5, over 50% of the liver cells were in the S phase, indicating that liver cells proliferate rapidly at these stages. At E17.5, the cell phase distribution started to shift from the S to G1 cell cycle phase, as demonstrated by a decrease in the S phase and an increase in the G1 phase (Figure 1A, B). Interestingly, at P14, more than 80% of the liver cells were in the G1 phase and, in adults, there was a further reduction in the percentage of cells in the S and G2/M phases (Figure 1A, B). Thus, liver cells seem to enter into a slow proliferation at stage of 2 to 3 weeks earlier than adult age. These data suggested that a reduction in the S phase and an expansion of the G1 phase constituted the main cell cycle shifts during the development of the mouse liver.

### Cell cycle drivers at different developmental stages

Next, we examined the expression of cell cycle drivers (cyclins and Cdks) in liver cells at different developmental stages. For the G1 and S phases, cyclin E and Cdk2, but not cyclin A, showed consistently high levels of expression at all developmental stages (Figure 1C). Cyclin D1 was expressed at low levels during most stages and was enhanced at P14 to P56, although the G1 cell phase was dominant at these developmental time points (Figure 1A, B, C). For the G2/M phase, cyclin B1 was consistently expressed at high levels during all stages and was further enhanced after the embryonic stage, whereas Cdk1 was down-regulated in the adult liver (Figure 1C). When the Cdk inhibitors were measured, p27 expression seemed to be in accordance with the increased G1 population, while p21 was not detectable (Figure 1D). Taken together, we observed that only Cdk1 and cyclin A were down-regulated during embryonic liver development.

### Establishment of the G1 checkpoint in E13.5/E15.5 embryonic liver cells

**Figure 2:**
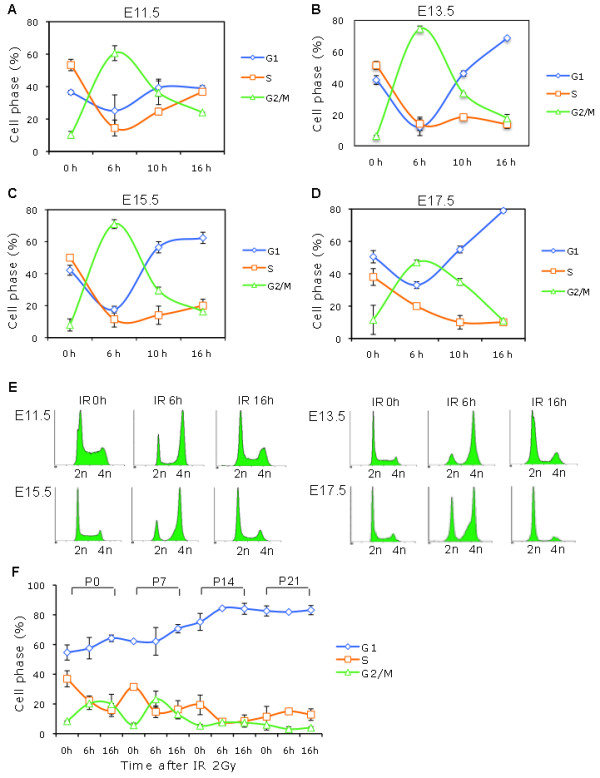
**Establishment of the IR-mediated G1 checkpoint in E13.5/E15.5 embryonic liver cells.** Pregnant mice at embryonic stages E11.5, E13.5, E15.5, and E17.5 were subjected to 0 or 6 Gy ionizing radiation (IR). At the indicated time points after IR, embryonic liver cells were isolated for PI staining and flow cytometric analysis. The cell cycle arrest patterns were determined at E11.5 **(A)**, E13.5 **(B)**, E15.5 **(C)**, and E17.5 **(D)** using Modfit software. Each analysis was on a cell pool of 3–5 embryonic livers. The data were the means ± SD from two independent analyses i.e. the embryonic liver cells were from different pregnant mice. Following transient G2 arrest, G1 arrest was observed at E13.5, E15.5, and E17.5 but not at E11.5. **(E)** Representative histograms of the cell cycle distribution patterns of E11.5, E13.5, E15.5, and E17.5 liver cells after 6 Gy IR at the indicated time points. **(F)** Mice were subjected to 0 or 2 Gy IR at different postnatal ages. Liver cells were isolated at the indicated time points for cell cycle analysis. Percentages of G1, S, and G2/M cells were determined. The mean ± SD was from 2 independent experiments.

It has been well demonstrated that ES cells are defective in the DNA damage-mediated G1 checkpoint [8-10]. At which developmental stage, and under which circumstances, the G1 cell cycle checkpoint is necessary for the cell is still unclear. By subjecting pregnant mice to IR, we were able to investigate the IR-mediated DNA damage checkpoint in liver cells from embryonic stages E11.5, E13.5, E15.5, and E17.5 to adulthood. In response to IR, liver cells showed a notable but transient G2 cell cycle arrest at E11.5, E13.5, E15.5, and E17.5, along with a significant reduction of G1 and S-phase cells (Figure 2A, B, C, D, E). At E11.5, the liver cells returned to a proliferating pattern, with the S phase population increasing at later time points following IR (Figure 2A, E). Starting at embryonic stage E13.5/E15.5, a significant G1 cell cycle arrest was observed, following a transient G2 arrest (Figure 2B, C, E). At E17.5, the extent of transient G2 arrest was reduced, whereas the IR-mediated G1 cell accumulation was more prominent (Figure 2D, E). From P0 to P21, the IR-mediated cell cycle arrest in all three phases was insignificant compared to that of the embryonic stage (Figure 2F) and showed a similar pattern as non-IR cells (Figure 1A). Thus, during early development, G2 arrest was the predominant DDR-mediated cell cycle arrest, and the G1 checkpoint appeared at the developmental stage of E13.5/E15.5.

### Cdk2 and p21 became regulated in response to IR in E13.5/E15.5 embryonic liver cells

**Figure 3:**
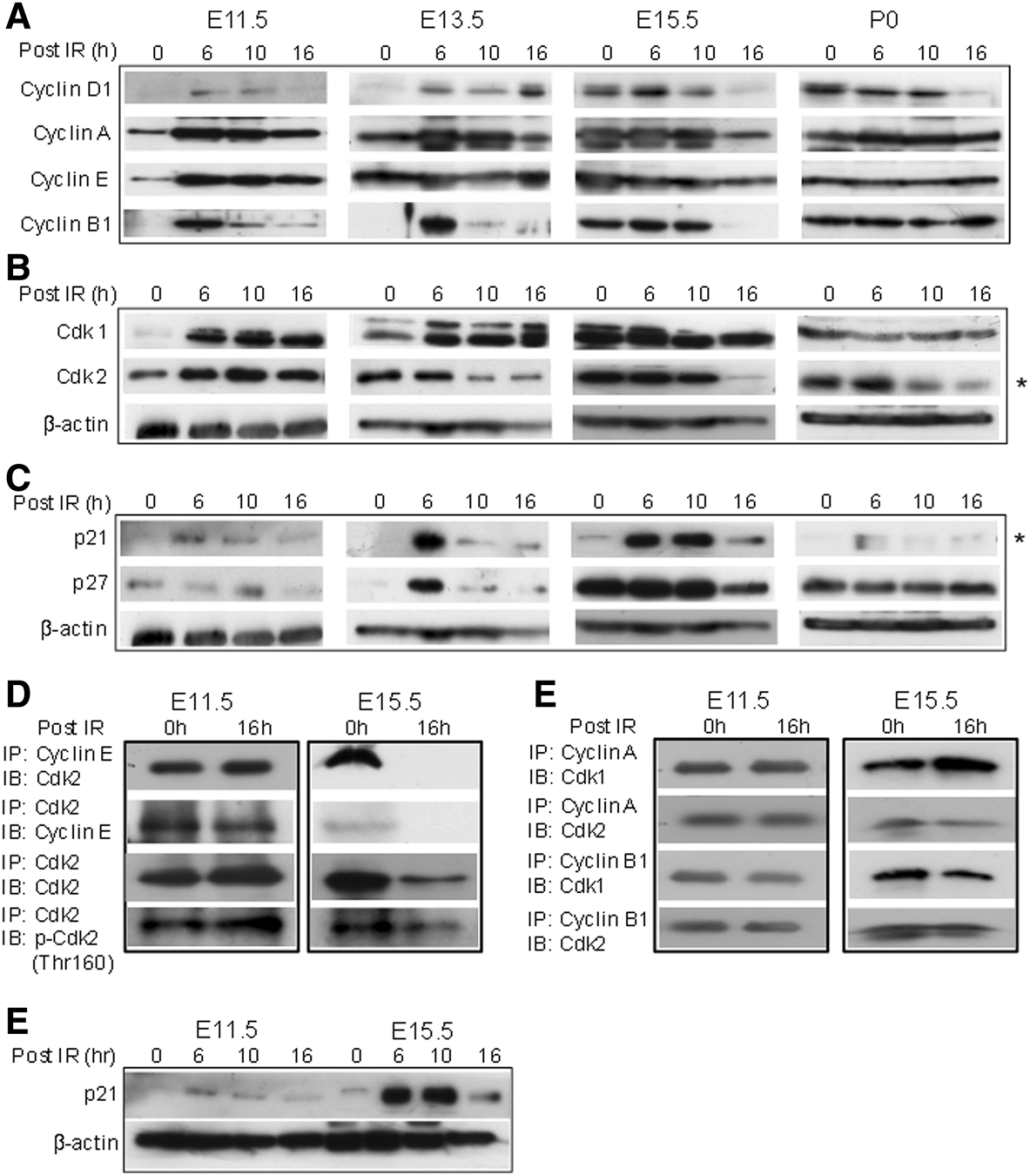
**The effects of Cdk2/cyclin E complex down regulation on the G1 checkpoint inE15.5 embryonic liver cells.** Pregnant mice were subjected to 0 or 6 Gy IR at E11.5, E13.5, E15.5 and P0. At the indicated time points after IR, embryonic liver cells were isolated for WB. **(A)** Protein levels of the cyclins D1, A, E, and B1. **(B)** Protein levels of Cdk1 and Cdk2. In response to IR, Cdk2 expression was down-regulated at E13.5 and E15.5. **(C)** Protein levels of p21 and p27. In response to IR, a dramatic induction of p21 was observed only at E13.5/E15.5. **(D)** The lysates from embryonic liver tissues (E11.5 and E15.5) harvested at 0 and 16 hours after IR were immunoprecipitated (IPed) with an antibody against cyclin E, and bound Cdk2 was detected by WB (left panel). The interaction pattern was representative of 2–3 experiments. To confirm the reduced expression of Cdk2/cyclin E complex, the lysates were IPed with an anti-Cdk2 antibody and then immunoblotted (IB) for cyclin E. The same lysates were IPed with anti-Cdk2 antibody and IBed with total Cdk2 and phospho-Cdk2 (Thr 160) (lower panel). **(E)** The lysates were IPed with antibodies against cyclin A and B1, and bound Cdk1 and Cdk2 were tested by IB. **(F)** IR-induced p21 expression was significantly enhanced in E15.5 liver cells compared to E11.5 cells.

We next investigated cell cycle checkpoint effectors that contribute to the formation of the G1 as well as G2/M checkpoint. These effectors, including cyclins D1, A, E, and B1, as well as Cdk1, and Cdk2, were evaluated at different time points before and after IR. In response to IR, Cdk2 was down regulated in E13.5/E15.5, but not in E11.5 embryonic liver cells (Figure 3A, B; highlighted by asters). No significant IR-mediated G1 checkpoint was observed at E11.5 (Figure 2A), suggesting that the down regulation of Cdk2 might be associated with the establishment of G1 cell cycle arrest. More importantly, the G1 checkpoint regulator p21 was enhanced to a greater level at E15.5 than at E11.5 (Figure 3C; highlighted by aster). No periodicity in accordance with cell cycle progression was observed with the other cyclins or with Cdk1 (Figure 3A, B).

### Regulation of the IR-mediated G1 checkpoint through the down-regulation of the Cdk2/cyclin E complex in E15.5 embryonic liver cells

The interaction of Cdks and cyclins in response to IR in liver cells was compared at E11.5 and E15.5 by co-IP. The expression of the Cdk2/cyclin E complex was significantly reduced after IR in E15.5, but not E11.5, liver cells (Figure 3D). This was confirmed by two different co-IP approaches (Figure 3D). Further, Cdk2 phosphorylation at Thr 160, which was necessary for the activation of Cdk2 complexes, was reduced after IR at E15.5 (Figure 3D). The interactions between Cdk1 and the cyclins A and B1 and between Cdk2 and the cyclins A and B1 remained the same before and after IR in both E11.5 and E15.5 liver cells (Figure 3E). These results suggested that reduced Cdk2 activity by the down regulation of phosphorylation of Cdk2 (Thr160) and the Cdk2/cyclin E complex was required for DNA damage-mediated G1 cell cycle arrest in E15.5 cells, and that this down regulation may be regulated by p21 (Figure 3F).

### Enhanced DNA damage repair capacity in E15.5 compared to E11.5 embryonic liver cells

**Figure 4:**
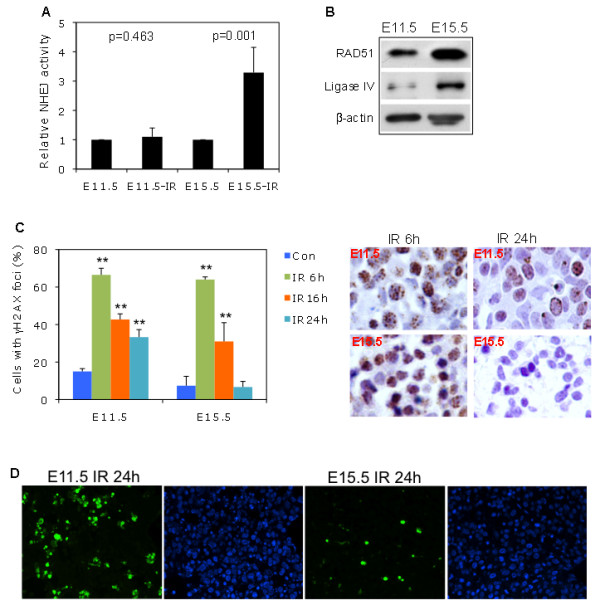
**High DNA damage repair capacity and low apoptosis in E15.5 compared to in E11.5 embryonic liver cells. (A)** In vitro NHEJ (non-homologous end-joining) activity was assayed and quantified by plasmid-based quantitative PCR (see Methods). Related NHEJ activity was analyzed by paired Student t test. IR-induced NHEJ activity was significantly higher at E15.5 than at E11.5. **(B)** Levels of related DNA repair proteins RAD51 and Ligase IV by WB. **(C)** Left panel: Pregnant mice were exposed to 6 Gy IR. At the indicated time points, E11.5 and E15.5 liver tissue was fixed for γH2AX foci (DSB foci) staining. Nuclear γH2AX foci-positive cells were enumerated. The mean ± SD was from 2 independent experiments. IR induced γH2AX foci-positive cells at different time points were statistically compared to controls, respectively. P value ≤0.05 and ≤ 0.01 was denoted as * and **, respectively. Right panel: Representative γH2AX staining showed that IR-induced DSB foci-positive cells were more dramatically reduced 24 hours after IR in E15.5 than in E11.5 liver cells. **(D)** Pregnant mice were exposed to 6 Gy IR. At 24 hours after IR, E11.5 and E15.5 liver tissue was fixed for TUNEL staining. Percentages of early apoptotic cells with nuclear FITC labeled nicked DNA vs. DAPI stained liver cells were calculated based on two sections in each case.

Murine ES cells have a higher DNA repair capacity than their differentiated derivatives such as fibroblasts [15]. We also compared the NHEJ pathway in E11.5 and E15.5 liver cells by an in vitro functional recombination assay using nuclear protein extracts. IR induced a 3-fold increase in NHEJ activity in E15.5 liver cells compared to baseline activity (p = 0.001) (Figure 4A), whereas this phenomenon was not observed in E11.5 liver cells (p = 0.463) (Figure 4A). The expression of the DNA repair proteins RAD51 (for HR) and Ligase IV (for NHEJ) seemed to be higher at E15.5 than E11.5 (Figure 4B). This finding suggested that there appeared to be more NHEJ repair at the developmental stage of E15.5 compared to earlier stages. HR activity could not be measured in this study, although studies have shown that NHEJ is a rapid and efficient repair mechanism [13,14].

### Short-term biological effects

In principle, the DNA damage-induced G1 cell cycle checkpoint would allow time for the repair of damaged DNA. Given that the G1 checkpoint was observed in E15.5 liver cells (Figure 2C) in combination with a significantly enhanced DNA damage repair activity (Figure 4A), we consequently asked whether this checkpoint might be biologically protective against DNA damage in the short and long term. We collected E11.5 and E15.5 embryonic liver tissue at 6, 16, and 24 hours after IR to evaluate the DNA damage foci. The number of γH2AX DSB foci containing nuclei initially increased to a similar extent (approximately 60%) in both E11.5 and E15.5 liver cells (Figure 4C, left panel). The γH2AX DSB foci positive cells remained significantly high at 24 hours after IR in E11.5 liver cells, but not in E15.5 liver cells. Thus, the amount of time required for the γH2AX foci to return to basal, non-IR levels (7.4%) was 24 hours in the E15.5 liver tissue. However, the percentage of DSB foci containing cells remained high (33%) in E11.5 liver tissue at this time point (Figure 4C, left and right panel). Previous studies have identified a linear association between γH2AX foci formation and DNA damage [16]. Therefore, the high percentage of γH2AX foci remaining in E11.5 liver tissue after 24 hours suggested sustained DNA damage at this developmental stage. We further determined cellular apoptosis and found a significantly higher percentage of early apoptotic cells in E11.5 liver tissue than in E15.5 liver tissue (Figure 4D, Table 1). Thus, E11.5 liver cells which lacking of G1 checkpoint also had a sustained DNA damage and increased cell death.

**Table 1:** Statistical comparison of early apoptosis in embryonic liver sections between E11.5 and E15.5 after IR

	**E11.5**	**E15.5**	***p *** **value**
**Early apoptosis (%)**	24.8% ± 1.7	6.7% ± 0.4	0.005

### Long-term biological effects

**Figure 5:**
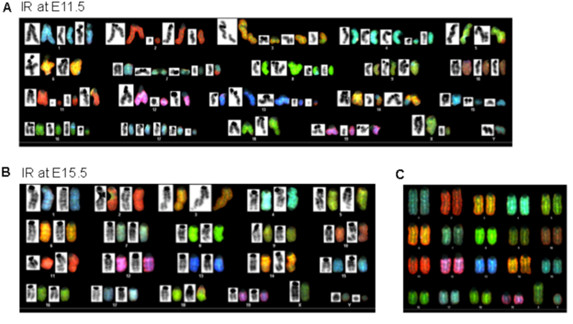
**Low dose IR at E11.5 and E15.5 and its effects on chromosome abnormality in adult liver cells. (A & B)** Pregnant mice were exposed to 0.5 Gy IR at embryonic stages 11.5 or 15.5, and the liver cells were then isolated from the mice at adulthood (7 weeks old) and cultured. Chromosome G-banding and spectral karyotyping (SKY) analysis was performed in metaphase cells. Overall aberrant chromosome at metaphase by G-banding (black color) and SKY (RBG color) in the adult liver cells IR at E11.5 **(A)** and at E15.5 **(B). (C)** Normal chromosome at metaphase by SKY.

For the evaluation of long-term effects, chromosome G-banding and SKY analysis were performed in murine adult liver cells derived from the embryos that were exposed to 0.5 Gy IR at E11.5 or E15.5. The chromosomes from 35 adult liver cells were examined in each case. The total number of chromosomes with breaks or rearrangements was significantly higher in adult liver cells derived from the embryos that were exposed to IR at E11.5 than at E15.5 (72 vs. 39, respectively) (Table 2). Amongst abnormal chromosomes, the percentages of chromosome breakage were over 80% in both groups of cells. However, the rates of deletion, which is one of the most severe forms of chromosome breakage, was significantly higher in the adult liver cells derived from the embryos that were exposed to IR at E11.5 (27%) than at E15.5 (6%). Figure 5A and B showed representative aberrant chromosome pattern at metaphase by G-banding and SKY analysis from liver cells derived from IR at E11.5 and E15.5 respectively, with overall chromosome abnormality looked more severe in liver cells IR at E11.5 (Figure 5A) than IR at E15.5 (Figure 5B) compared to a normal chromosome pattern (Figure 5C). Chromosome endoreduplication phenomenon was also observed in the adult liver cells derived from the embryos that were exposed to IR at E11.5 but not at E15.5 (Additional file 1: Figure S2; pointing by yellow arrows), which might result from abnormal mitosis leading to cellular polyploidy. Moreover, postnatal deaths occurred in response to 2 Gy IR at E11.5 but not at E15.5 (data not shown). Thus, G1 checkpoint initiation and enhanced DNA repair might be able to reduce mortality and severe chromosome damage in the long term.

**Table 2:** G-banding & spectral karyotyping (SKY) analysis in adult liver cells born from mouse exposing to IR (0.5 Gy) at E11.5 or E15.5 embryonic stage

**Chromosome abnormalities**	**IR at E11.5**	**IR at E15.5**
Overall abnormal chromosomes	72	39
Chromosome breakage (%)	83	82
Chromosome rearrangement (%)	17	18
Chromosome deletion among breakage (%)	27	6

Legend: Total of 35 adult liver cells were examined in IR at E11.5 and E15.5 respectively, with full chromosome karyotyping of each cells. 15 out of 35 liver cells contained abnormal chromosomes in both E11.5 and E15.5. Comparisons of overall number of chromosomes with breakage or rearrangements and severe abnormal chromosome sub-categories between E11.5 and E15.5 were summarized in Table 2.

## Discussion

In this study, we characterized the establishment of the G1 checkpoint and the associated DSB repair, as well as the biological effects of these processes, in the murine embryonic liver at different stages of development. It is the first investigation using live embryonic liver tissue to investigate the DNA damage-mediated checkpoint and repair during embryonic development.

ES cells lack DNA damage-mediated G1 checkpoints in culture [8-10] and employ S and G2 cell cycle arrest as protective mechanisms [9,10]. When ES cells differentiated and entered into lineage and organ development, G2 cell cycle arrest was still the earliest response to DNA damage (Figure 2A, B, C, E) [6] during the early developmental stages of embryonic liver cells (E11.5 to E15.5). Beginning at E13.5/E15.5, the embryonic liver cells started to establish a G1 DNA damage checkpoint, where G1 cell cycle arrest replaced transient G2 arrest for longer periods of time following DNA damage (Figure 2A, B, C, D, E) [6]. It is unclear why the G1 checkpoint is established so much later than the G2 checkpoint. A requirement for rapid proliferation results in the cells being mostly in the S and G2/M cell cycles, with a short G1 phase, during the early developmental stage, which might also equip cells with S and G2/M cell cycle checkpoint mechanisms in response to DNA damage. During the differentiation of ES cells and embryo development, the G1 cell cycle length and G1 population both increase [4-7,17-19], as does the G1 checkpoint machinery (Figures 1A, 2A, B, C, D) [6]. More importantly, cell cycle arrest provides time for the repair of DNA damage. The G2 arrest could prevent apoptosis, while the G1 arrest is important to prevent damaged DNA from entering into the S phase. Thus, by comparison between E11.5 and E15.5, our study demonstrates that the development of the G1 checkpoint play a critical role in long-term genome stability (Table 2) (Figure 5A, B), although aberrant chromosomes still exist in the adult liver cells, and protection is, therefore, not complete (Table 2).

**Figure 6:**
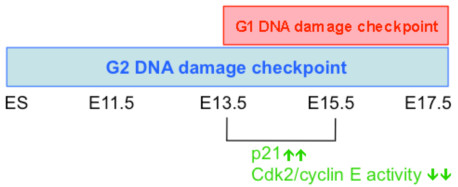
**A proposed model of G1 checkpoint establishment during embryonic liver development.** In response to IR damage, G2 cell cycle checkpoint is present in ES cells [8-10] and in E11.5 to E17.5 embryonic liver cells. At E13.5/E15.5 stage, G1 cell cycle checkpoint is established under the regulation of p21-CDK/cyclin E pathway.

Clearly, the initiation of the G1 checkpoint in E13.5/E15.5 embryonic liver cells was regulated by the declining activity of the Cdk2/cyclin E complex (Figure 3D, C, E). The down-regulation of Cdk2 did not appear to be regulated by p27, as the p27 expression levels were associated with an increased G1 population during embryonic liver development, regardless of the DDR (Figures 1D, 3C). However, p21 expression was not related to the G1 population expansion during embryonic liver maturation (Figure 1D) but was instead induced by DNA damage (Figure 3C, F), and induced p21 expression was more robust at E13.5/E15.5 than at earlier stages (Figure 3F). The association between differentiation and the length of the G1 phase has always been a topic of interest. Recently, a comprehensive study demonstrated that in the process of murine ESC differentiation, p21 accumulation (under regulation of mir-290 miRNA) results in the inactivation of Cdk2 and cyclin E, leading to a delay in the G1-to-S phase transition; these results suggest interdependence between G1 length and differentiation [18,19]. Albeit not restricted to ES cells, G1 length can directly impact the differentiation of neural and hematopoietic stem cells during development and adulthood [6,20], of which, down-regulation of Cdk2/cyclin E seems to be one of the main mechanical drives in G1 lengthening [20]; both inhibition of Cdk2/cyclin E activity [21] or depletion of Cdk2/Cdk4 [22] resulted in increasing G1 length and consequently neuronal differentiation. In embryonic liver, our study expends the role of down-regulation of Cdk2/cyclin E in DNA damage mediated G1 cell cycle arrest (Figures 2, 3 and 4). Thus, the proposed regulatory mechanism for G1 phase expansion in mESC or in neuronal SC differentiation also applied to the initiation of the G1 DNA damage checkpoint during embryonic liver cell development. The G1 checkpoint only appeared at the stage (E13.5/15.5) where IR induced high expression of p21, which led to the inhibition of the Cdk2/cyclin E complex and the block in the G1-to-S transition (Figures 2, 3 and 6). Thus, p21-Cdk2/cyclin E-mediated G1 expansion [18,19,4] seems to be a common regulatory mechanism for both the length of the G1 cell phase and the DNA damage-mediated G1 cell cycle arrest (Figure 6).

γH2AX positive cells in E11.5 livers remained high throughout longer time course after IR (Figure 4C), the relative larger population of E11.5 liver cells in S phase could be a cause; as it has shown that DSB foci developed during the course of the UV-induced replication arrest [23]. However, a minority of the UV induced foci showed DSB γH2AX colocalizing with 53BP1, whereas the majority was pan-nuclear γH2AX, a pre-apoptotic signal in the S phase [24]. In our experimental setting: (a) IR did not induce replication arrest (Figure 2); (b) after a short G2 arrest, E11.5 liver cells returned to a basal level but not an S phase arrest (Figure 2); (c) the fraction of γH2AX positive cells was not significantly different between E11.5 and E15.5 liver cells under the condition without IR (p = 0.349) (Figure 4C), whereas S phase population of both were high (over 50%) (Figure 2A, C). Moreover, rapid NHEJ repair is not limited to specific cell phase but occurs throughout all cell cycle phases [25]. Thus, high levels of γH2AX foci in E11.5 liver cells were likely due to their DNA repair capability not being competent enough, as well as lacking G1 arrest checkpoint control (Figures 2 and 4). In accordance, early apoptosis was significantly higher in E11.5 compared to E15.5 liver cells (p = 0.005) (Figure 4D, Table 1), which is a cellular consequence of absence of G1 checkpoint as well as retaining high level of un-repaired DNA damage in E11.5.

## Conclusion

The initiation of the DNA damage-mediated G1 cell cycle arrest occurs at embryonic stage E13.5/E15.5 in embryonic liver cells, and this process is regulated by the p21-mediated down-regulation of the Cdk2/cyclin E complex. The G1 checkpoint, in combination with DNA repair, plays a biological role in repairing cellular DSBs and in preventing early apoptosis in the short term and reducing chromosome abnormalities in the long term.

## Competing interests

The authors declare that they have no competing interests.

## Authors’ contributions

XQW conceived and designed the study, carried out the gene expression study and draft manuscript. KKC carried out embryonic liver cell cycle study. XM participated in the DNA repair study. VCL participated in the design of the study and helped to draft the manuscript. RYP, CML, CN, and RTP helped in the design of the study and provide materials. All authors read and approved the final manuscript.

## Supplementary Material

Additional file 1: Table S1 Primers for *in vitro* NHEJ assay. **Figure S1.** (A) Morphologies of mouse livers from embryonic, postnatal and adult stage were analyzed by H&E staining. (B) Flow cytometry analysis of albumin expression in embryonic liver. Single cell suspension of embryonic livers was stained with antibody specific for albumin or non-immune IgG. The albumin-positive cell population was gated with reference to IgG control (left panel), and shown as histogram (right panel). **Figure S2.** Pregnant mice were exposed to 0.5 Gy IR at embryonic stages 11.5 and 7 week adult liver cells were isolated and cultured. Chromosome G-banding (A) and spectral karyotyping (SKY) (B) analysis was performed in metaphase cells. Yellow arrows pointed to the images of abnormal chromosome endoreduplication. Click here for file
