# Supplementary material for: G1 checkpoint establishment in vivo during embryonic liver development
Source: BMC Dev Biol. 2014 May 19;14:23. doi: 10.1186/1471-213X-14-23 (PMC4031160; doi:10.1186/1471-213X-14-23)

Table S1. Primers for *in vitro* NHEJ assay and mouse gene expression

Primers	Forward primer (F)<br>Reverse primer (R)
Set A (loading control)	F: 5’-GGTGATGACGGTGAAAACCT-3’
	R: 5’-GCTCTGATGCCGCATAGTTA-3’
Set B (joining junction)	F: 5’-GGAGAAAATACCGCATCAGG-3’
	R: 5’-GAGTCAGTGAGCGAGGAAGC-3’

Figure S1

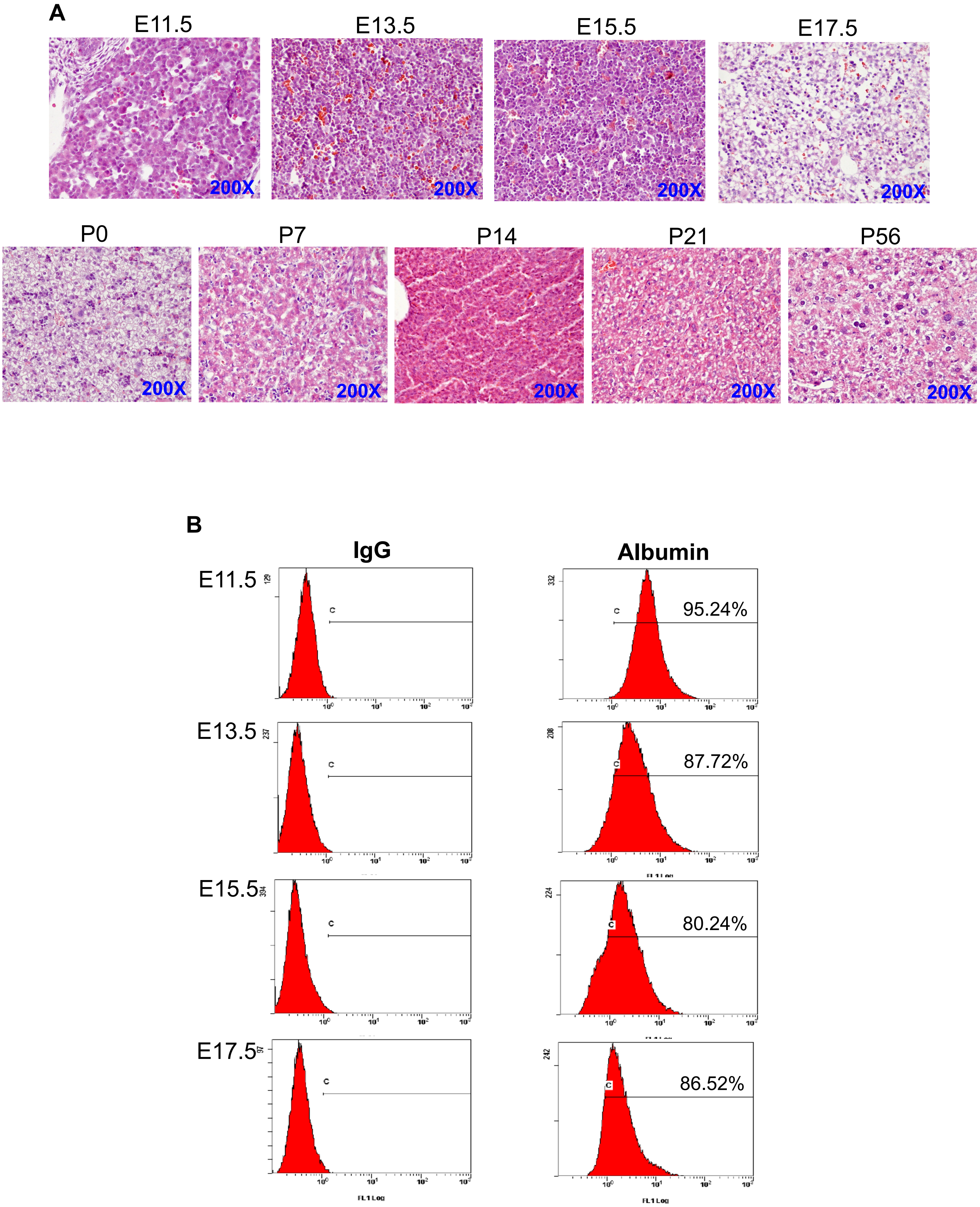
A
E11.5
E13.5
E15.5
E17.5
200X
P0
P7
P14
P21
P56
B
IgG
Albumin
E11.5
E13.5
E15.5
E17.5
95.24%
87.72%
80.24%
86.52%
FL1 Log

Figure S2

A

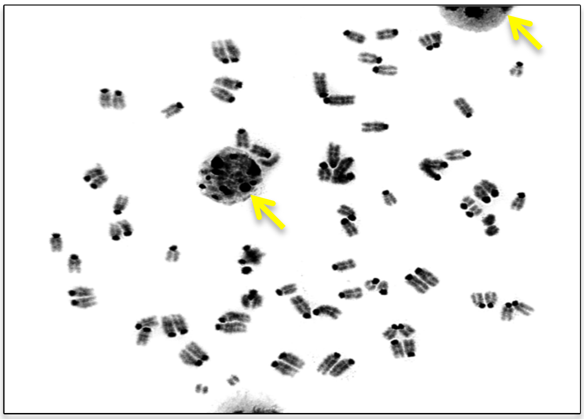

B

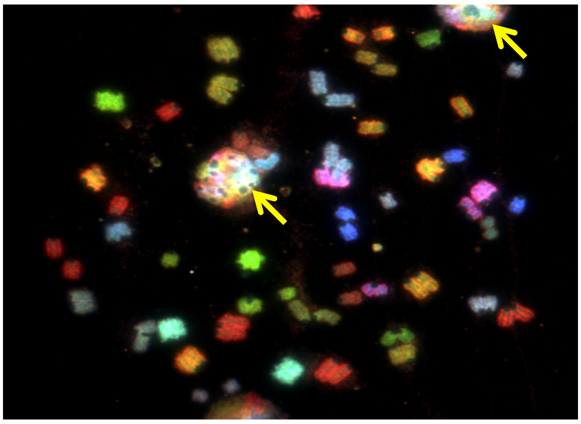

Supplement: Additional file 1: Table S1 — Primers for in vitro NHEJ assay. Figure S1. (A) Morphologies of mouse livers from embryonic, postnatal and adult stage were analyzed by H&E staining. (B) Flow cytometry analysis of albumin expression in embryonic liver. Single cell suspension of embryonic livers was stained with antibody specific for albumin or non-immune IgG. The albumin-positive cell population was gated with reference to IgG control (left panel), and shown as histogram (right panel). Figure S2. Pregnant mice were exposed to 0.5 Gy IR at embryonic stages 11.5 and 7 week adult liver cells were isolated and cultured. Chromosome G-banding (A) and spectral karyotyping (SKY) (B) analysis was performed in metaphase cells. Yellow arrows pointed to the images of abnormal chromosome endoreduplication. [file 1471-213X-14-23-S1.pdf]
